# Consistent Assignment of Risk and Benign Allele at rs2303153 in the CF Modifier Gene *SCNN1B* in Three Independent F508del-*CFTR* Homozygous Patient Populations

**DOI:** 10.3390/genes12101554

**Published:** 2021-09-29

**Authors:** Frauke Stanke, Tim Becker, Haide Susanne Ismer, Inga Dunsche, Silke Hedtfeld, Julia Kontsendorn, Anna-Maria Dittrich, Burkhard Tümmler

**Affiliations:** 1 Clinic for Pediatrics, Department of Pediatric Pneumology, Allergology and Neonatology, Hannover Medical School, 30625 Hannover, Germany; Haide.S.Ismer@stud.mh-hannover.de (H.S.I.); Inga.Dunsche@stud.mh-hannover.de (I.D.); Jansen.Silke@mh-hannover.de (S.H.); Kontsendorn.Julia@mh-hannover.de (J.K.); Dittrich.Anna-Maria@mh-hannover.de (A.-M.D.); tuemmler.burkhard@mh-hannover.de (B.T.); 2 Biomedical Research in Endstage and Obstructive Lung Disease Hannover (BREATH), The German Center for Lung Research (D.Z.L.), 30625 Hannover, Germany; 3 Institute for Community Medicine, Ernst Moritz Arndt University Greifswald, Germany & xValue GmbH, 47877 Willich, Germany; tim.becker@uni-greifswald.de

**Keywords:** cystic fibrosis, association study, modifier gene, amiloride-sensitive sodium channel ENaC

## Abstract

*CFTR* encodes for a chloride and bicarbonate channel expressed at the apical membrane of polarized epithelial cells. Transepithelial sodium transport mediated by the amiloride-sensitive sodium channel ENaC is thought to contribute to the manifestation of CF disease. Thus, ENaC is a therapeutic target in CF and a valid cystic fibrosis modifier gene. We have characterized *SCNN1B* as a genetic modifier in the three independent patient cohorts of F508del-*CFTR* homozygotes. We could identify a regulatory element at *SCNN1B* to the genomic segment rs168748-rs2303153-rs4968000 by fine-mapping (Pbest = 0.0177), consistently observing the risk allele rs2303153-C and the contrasting benign allele rs2303153-G in all three patient cohorts. Furthermore, our results show that expression levels of SCNN1B are associated with rs2303153 genotype in intestinal epithelia (*p* = 0.003). Our data confirm that the well-established biological role of SCNN1B can be recognized by an association study on informative endophenotypes in the rare disease cystic fibrosis and calls attention to reproducible results in association studies obtained from small, albeit carefully characterized patient populations.

## 1. Introduction

Cystic fibrosis (CF) is caused by two defective copies of the cystic fibrosis transmembrane conductance regulator gene *CFTR* and is considered the most common severe monogenic disease inherited in an autosomal recessive fashion among the population of Western European ancestry [1]. *CFTR* encodes for a chloride and bicarbonate channel expressed ubiquitously at the apical membrane of polarized epithelial cells, causing a generalized exocrinopathy [1]. Transepithelial sodium transport mediated by the amiloride-sensitive sodium channel ENaC is thought to contribute to the manifestation of CF disease. Thus, ENaC is a therapeutic target in CF [2,3].

The gene *SCNN1B* encodes the beta-subunit of the amiloride-sensitive sodium channel ENaC [4]. *SCNN1B* is known to cause Liddle syndrome [5] (OMIM #177200), pseudohypoaldosteronism [6] (OMIM #264350), and to contribute to the manifestation of bronchiectasis [7,8] (OMIM #211400) and CF-like disease [8]. Furthermore, *SCNN1B* has been described as a modifier of CF in humans [9,10] and a murine model hyperexpressing Scnn1b shares several aspects of the pathophysiology with CF [11].

While many CF modifying genes beyond *SCNN1B* have been described (reviewed by [12,13]), ENaC’s subunits are biologically plausible candidates which have hitherto escaped notice in genome-wide screens [14,15] and in systematic multi-omics data mining approaches [16]. In that line, missing heritability has been recognized and accepted for other gene loci in polygenic diseases where many loci might each have a small impact on the phenotype under study [17,18]. The size of the population under study as well as the threshold accepted for significance will determine the ratio of true-positive findings and false-positive findings of loci with a small impact [19], demanding replication studies that inspire another set of complications [20].

Typically, genes queried in studies that maximize their study population in order to achieve higher power. However, for cystic fibrosis, large cohorts are predisposed to confounders in several ways. CF is characterized by allelic heterogeneity. More than 2000 variant *CFTR* genes have been described in the context of CF disease. In the population of mid- and central European ancestry, 70% of *CFTR* alleles are F508del-*CFTR*, making it feasible to study genetic modifiers in a patient population that shares their disease-causing mutation. Moreover, continuously improving treatment regimens have steadily increased patient survival and quality of life in recent decades [21]. As a consequence, the desire to recruit many patients into a study for genetic modifiers will lead to a mixture of recently born patients with an older population of people with CF, whereby not all individuals from a particular birth cohort can be enrolled due to the survivor effect. In conclusion, the replication of genetic findings require that the study populations are similar with respect to major non-genetic determinants that influence the phenotype. Consequently, different degrees of heterogeneity, e.g., in birth cohort, in the distribution of socioeconomic status, or other non-inherited determinants, might explain why studies on CF modifying genes have disagreed in the past [22,23,24].

In this work, we present data on rs2303153 from three different association studies. We have employed recruitment strategies to minimize the effect of non-genetic determinants and have assessed the survivor effect that has been noticed for other CF modifying genes before [23,25,26]. We could verify that the putative causative SNP rs2303153 in *SCNN1B* shows an allelic association to SCNN1B transcript levels in patients’ tissue. In conclusion, we present data which indicate that rs2303153 influences the course of CF disease by regulating *SCNN1B* expression, building on non-overlapping, small, albeit carefully described patient samples.

## 2. Materials and Methods

### 2.1. Patient Cohorts

*SCNN1B* was characterized as a genetic modifier in the following three independent patient cohorts of F508del-*CFTR* homozygotes: 1. twins and siblings with cystic fibrosis from European CF centres whereby disease severity was highly similar within a pair; 2. twins and siblings with cystic fibrosis from European CF centres whereby disease severity was highly dissimilar within a pair; 3. unrelated CF patients recruited from the local CF center at Hannover. The European CF twin and sibling study was approved by the ethics committee of Hannover Medical School and written informed consent was obtained from all participants or their parental guardians (ethics committee vote #2771). The study on unrelated patients from the CF center in Hannover was conducted under the ethics committee vote #3739. All methods were performed in accordance with relevant guidelines and regulations.

For the European CF twin and sibling study, case and reference populations with extreme clinical phenotypes were selected as described elsewhere [25,27]. Briefly, data on weight, height and forced expiratory volume within one second (FEV1) were collected for more than 300 CF twin and sibling pairs. Anthropometry data were converted to weight as % of predicted weight for height. Lung function data on FEV1 was converted to % of expected values for a non-CF population and expressed as CF population centiles for FEV1%pred. Centiles for these parameters were age-independent among the recruited CF twin and sibling pairs [27]. To describe CF disease severity for each patient in the two major affected organ systems, a composite parameter was defined based on rank numbers for weight as % of predicted weight for height as well as for CF population centiles for FEV1%pred. Next, a ranking algorithm was used to describe intrapair discordance as well as disease severity of all sibling pairs. Finally, by this ranking algorithm, the most mildly affected and concordant pairs, the most severely affected and concordant pairs, and the most discordant pairs composed of one mild and one severely affected sibling were identified. For genetic analysis, 14 discordant (DIS), 11 concordant mildly (CON+), and 10 concordant severely (CON−) affected sibling pairs were selected out of 114 F508del-*CFTR* homozygous sibling pairs [27]. In this sibling pair population, the effect of non-inherited confounders is minimized as two siblings share many environmental determinants such as access to therapeutic care or the socioeconomic status during childhood. Furthermore, we have defined extreme contrasting phenotypes to increase the sensitivity of our study [28,29].

From the CF clinic in Hannover, we conducted a retrospective longitudinal study which builds upon clinical data collected between 1985 and 2015 of more than 297 patients. Clinical data were retrieved from the data archive used to collect clinical data since 1977. Hence, 140 patients for which DNA could be recovered from the local biomaterial bank were included into the study. We have previously reported data of this cohort on patient subsamples for *TGFB1* [23] and on *IL1R* [26] whereby we could describe a survivor effect among early-born patients that manifest as an enrichment of mild alleles at these two CF modifying genes [23,26]. Here, we have revisited this CF population and we have used the year of birth and the initially recorded lung function to assess disproportionate allele distributions among these patients that can arise if not all individuals from a particular birth cohort can be enrolled due to the survivor effect and if survival is dependent on the gene under study.

### 2.2. SCNN1B Genotyping

The genotyping data set described here has been employed within two projects: 1. within this work, to describe the regulatory element *in cis* that directly influences *SCNN1B* expression and 2. to describe a regulatory element that is targeted by DNA binding proteins which are encoded *in trans* [10]. These two approaches describe two different intragenic elements, both of which are relevant to understand the CF modifying gene *SCNN1B*. In particular, our previous work [10] was recognized by comparing discordant sibling pairs to all concordant sibling pairs at SNPs rs152730-rs152731-rs152745-rs152744-rs152741-rs152740 which span an area from exon 2 to exon 4 in SCNN1B. In contrast, this work on rs2303153 between exon 11 and exon 12 describes a regulatory element in that was recognized directly by comparing mildly to severely affected patients and sibling pairs. Exon numbers in *SCNN1B* refer to: [30].

The genotyping data set is described in detail elsewhere [10]. Briefly, to map the previously observed association signals within the *SCNN1B/SCNN1G* genomic region on European CF twins and siblings [9], 48 SNPs were selected based on predicted informativity (MAF > 0.4) and position on the genomic map, aiming for a spacing of 2 kb to 10 kb between adjacent SNPs. In the SNPstream high-throughput genotyping apparatus (Beckman Coulter Inc., Brea, CA, USA), genotyping was carried out in a multiplex SNPstream assay. Markers for which no information could be obtained were retyped by PCR-RFLP on the entire collection of more than 300 DNA samples. Furthermore, rs168748 was genotyped by PCR-RFLP to avoid an intramarker gap of more than 10 kb between adjacent informative SNPs.

Genetic information on the cohort of unrelated CF patients from the CF centre in Hannover was obtained only for rs2303153 by PCR-RFLP using primers 5′AGTTTGGACACAGGACAGCT and 5′ATGCACAGTGACAGAGGGAA. PCR products were digested with *ScrF*I which recognizes the allele rs2303153-G.

### 2.3. Evaluation of Genetic Data

For data obtained from the European CF twin and sibling study, we have analyzed the marker set for an association with disease severity by comparing allele- and haplotype distributions between concordant mildly affected siblings and concordant severely affected siblings as well as between mildly affected sibs of discordant pairs and severely affected sibs of discordant pairs. Genetic data on European CF twins and siblings were evaluated using the FAMHAP software package [31]. FAMHAP allows family-based analysis [32,33], accepts data evaluation in association studies on unrelated individuals as well as on affected sib pairs, and was adapted to handle intrapair comparison of genotype data in sib pairs [31]. The analysis of more than one marker per locus was corrected for multiple testing by haplotype permutation [33], whereby permutation was done by randomly assigning the affection status to the individuals in each replication [31].

Data obtained on rs2303153 on unrelated CF patients from the clinic in Hannover was analysed by directly comparing genotype and allele frequencies between subgroups stratified by birth cohort using compared using Monte Carlo simulation with CLUMP [34].

### 2.4. Retrieval and Analysis of SCNN1B Expression Data

Data on *SCNN1B* expression were retrieved from a transcriptome analysis conducted on unrelated F508del-*CFTR* homozygotes on rectal suction biopsies used for intestinal current measurement from a previous study [25,35]. These biosamples mainly represent intestinal epithelium. The corresponding data set and its metadata were deposited in the GEO database under accession number GSE15568. The expression data for 22283 probe sets were normalized and evaluated using Affymetrix Microarray Suite v5.1 software. For *SCNN1B* expression, data on probe set 205464_at were extracted. Expression levels between carriers of GG, CG, and CC genotypes were compared by a *t*-Test.

### 2.5. WWW Resources

Genomic sequences were retrieved from the NCBI database (https://www.ncbi.nlm.nih.gov/search/ accessed on 24 March 2021). Predicted sites for the general transcription factor TFII-I were observed using HaploReg (http://compbio.mit.edu/HaploReg; [36]; accessed on 24 March 2012). ORegAnno annotation was retrieved from the UCSC genome browser (http://genome.ucsc.edu/; [37]; accessed on 23 June 2021).

## 3. Results

### 3.1. Mapping of a Regulatory Element at SCNN1B to the Genomic Segment rs168748-rs2303153-rs4968000

We have used informative markers (MAF > 0.4) to describe haplotype blocks (D’ > 0.8) and map association signals by these adjacent ancestral informative markers as previously described for a the CEACAM gene cluster on chromosome 19 [38]. Briefly, for haplotype-based iterative fine-mapping, we assume that the fragment that carries the causative variant(s) can be recognized by Pbest in the association signals for two-marker-haplotypes defined by ancient SNPs that may have occurred prior to the causative SNP. We further assume that such ancient SNPs are recognized by a high minor allele frequency and that the causative variant that we look for is in LD with both adjacent markers defining such 2-marker-haplotypes. Locus-spanning 20-marker-haplotypes were reconstructed based on a set of informative markers (observed MAF > 0.4). The genotype data of 101 families with a total of 171 patients from the European CF twin and sibling study was provided as a training set to ensure a consistent assignment of rare haplotypes in these small non-overlapping subsamples.

**Figure 1:**
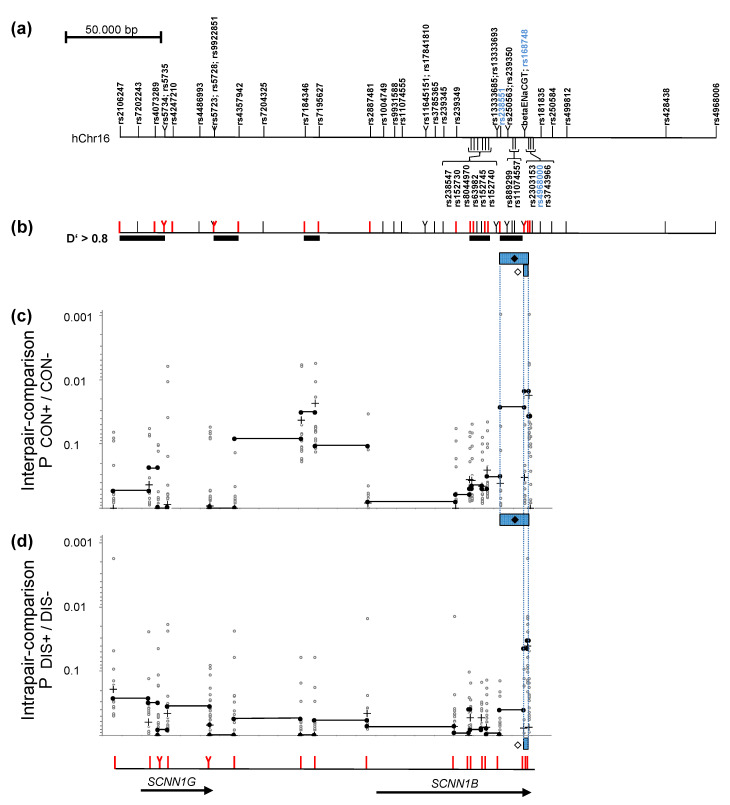
Mapping of a regulatory element within *SCNN1B* using an association study in two non-overlapping sets of affected patent pairs (**a**) Genomic map of the SCNN1B/SCNN1C region analysed by 7 previously typed markers (Stanke et al., 2006) and 49 SNPs genotyped for fine-mapping. Markers rs238551, rs168748 and rs4968000 are highlighted in blue as they define the fragments of interest (see (**c**,**d**)). (**b**): Markers depicted in red are informative (MAF > 0.4) and were used to describe haplotype blocks (D’ > 0.8; black bars) and map association signals for interpair-comparison of concordant mildly (CON+) and concordant severely (CON−) affected patient pairs (**c**) and for intrapair comparison of mildly (DIS+) and severely (DIS−) affected siblings within discordant pairs (**d**). (**c**,**d**): Association signals comparing CF patients with contrasting phenotypes. *p*-values corrected for multiple testing of 20 informative markers were: Pcorr CON+/CON− = 0.0528 and Pcorr DIS+/DIS− = 0.05. Uncorrected raw P values are shown for single markers (+), 2-marker-haplotypes of adjacent informative markers (filled circles in all diagrams; genomic segments spanned by two adjacent markers are linked by a black line) and 2-marker-haplotypes of non-adjacent markers (open circles). (**c**): Interpair comparison of concordant sibling pairs: P values were computed comparing haplotype distributions of 11 concordant mildly affected patient pairs (CON+) and 10 concordant severely affected patient pairs (CON−). The fragment marked ♦ corresponds to rs238551 to rs4968000. (**d**): Intrapair comparison within discordant pairs. P values were computed comparing the mildly affected sibling (DIS+) to the severely affected sibling (DIS−) for 14 discordant pairs. The fragment marked ♢ corresponds to rs168748 to rs4968000.

For the analysed *SCNN1B/SCNN1G* region, we have observed an association signal comparing 10 concordant severely affected sib pairs to 11 concordant mildly affected sib pairs, defining interpair association with disease severity (Figure 1; Pbest = 0.00094 for rs238551-rs2303153; Pcorr = 0.0528 corrected for multiple testing of 20 informative markers) and an association signal comparing mildly and severely affected sibling of 14 discordant patient pairs (Figure 1; Pbest = 0.00174 for rs2106247-rs2303153; Pcorr = 0.05 corrected for multiple testing of 20 informative markers), defining intrapair association with disease severity. Fragments carrying the causative variant(s) (Figure 1) were assigned based on best Praw values observed for case reference comparisons of 2-marker-haplotypes from adjacent informative markers. The association signal observed for “disease severity” was seen on fragments rs238551-rs168748 (Praw = 0.02716), rs168748-rs2303153 (Praw = 0.01522) and rs2303153-rs4968000 (Praw = 0.03713) for the interpair comparions (Figure 1; fragment ♦) and on fragments rs168748-rs2303153 (Praw = 0.04494) and rs2303153-rs4968000 (Praw = 0.03371) for the intrapair comparison (Figure 1; fragment ♢). Fragments associated with disease severity based on the interpair comparison (♦) and on the intrapair comparison (♢) match for the genomic segment rs168748-rs2303153-rs4968000 and while the patient subsamples for these two independent comparisons are non-overlapping by definition [27].

The genomic sequence of rs168748-rs4968000 was ascertained by Sanger sequencing whereby homozygotes for contrasting haplotypes were compared to capture all genetic variants residing on these genomic fragments. Primers 5′CAGATCACTTGATGCCAGGA and 5′ GAAATTCAAATTCAACCAAGCAG were used to amplify a 4962 bp product for homozygous carriers of rs168748-rs2303153-rs4968000 haplotypes CCA, TCA, TGC and TGA. Samples defined by rs2303153 differed at five positions, i.e., at rs62029389-rs62029390-rs62029391-rs61379932-rs2303153 with GGGGC observed on the risk allele and ATAAG observed on the mild allele (Table 1), spanning 1289 bp between rs62029389 and rs2303153. rs2303153 is not in LD with the neighboring markers rs168748 located 2 kb on the 5′ side (*r*^2^ = 0.086; D’ = 0.341) and rs4968000 located 1 kb on the 3′ side (*r*^2^ = 0.070; D’ = 0.277).

In summary, the genomic fragment associated with disease severity can be recognized on rs168748-rs2303153-rs4968000 by an overlapping fragment when comparing mildly and severely affected patients in two non-overlapping sibling subgroups. rs2303153 displayed the most pronounced allelic association in both sibling subsample.

### 3.2. Assessment of the Risk and the Benign Allele at rs2303153

As the genomic position for the putative causative SNP in *SCNN1B* was consistently identified in two case reference comparisons conducted on sibling pairs of the European CF twin and sibling study, we next wanted to know which of the two rs2303153 alleles confers the risk to the CF patients.

Annotation of benign and risk allele at rs2303153 was consistent for the intrapair- and the interpair association as the benign allele rs2303153-G is more frequent among CON+ and DIS+ patients (Table 2). Allele C at rs2303153 was overrepresented among severely affected patient pairs, both in the intrapair and the interpair comparison (Table 1), indicating that rs2303153-C is a risk allele. Both neighboring markers are not in LD with rs2303153 (D’ = 0.341 for rs168748-rs2303153 and D’ = 0.277 for rs2303153-rs4968000), indicating the causal variant is close to rs2303153 on a very small LD block.

Furthermore, we reviewed data on rs2303153 from our CF centre in Hannover whereby lung function and year of birth were used to define patient subsamples. For patients who displayed a lung function below the median within this population of unrelated F508del-CFTR homozygotes, genotype distribution at rs2303153 was different from the distribution expected under the Hardy–Weinberg law, which indicates that alleles at 2303153 have been under selection pressure within this patient cohort. For patients who had a lung function above median, allele rs2303153-G was more frequent among patients born 1959 to 1977, indicating again that rs2303153 is mild among those survivors from whom DNA was sampled in the mid-nineties.

### 3.3. Functional Annotation of the Genomic Element Surrounding rs2303153

rs2303153 resides on the regulatory element OREG1500477 [37] and thus we asked whether gene regulation mediated by rs2303153 can be understood using in silico annotation. Curiously, rs62029389, rs62029390, and rs62029391 correspond to nearly adjacent positions within a complex CT-rich repeat (Supplementary Figure S1), potentially disrupting the secondary and tertiary structure of the DNA by introducing three nucleotides capable to form three hydrogen bonds at rs62029389, rs62029390, and rs62029391 on haplotype GGGGC at rs62029389-rs62029390-rs62029391-rs61379932-rs2303153 in comparison to three nucleotides capable to form two hydrogen bonds at rs62029389, rs62029390, and rs62029391 on haplotype ATAAG at rs62029389-rs62029390-rs62029391-rs61379932-rs2303153. Furthermore, the repetitive element encountered near rs62029389, rs62029390, and rs62029391 has the potential to form triple-helical DNA and H-DNA structures [39] and such structures have been suspected to have a regulatory role [40,41,42] Finally, we have used Haploreg [36] to ask whether SNPs on the analysed genomic segment alter regulatory motifs. rs62029389, rs62029390, and rs62029391 disrupt a binding site for the general transcription factor TFII-I (Supplementary Figure S2). In summary, for the genomic fragment associated with disease severity by interpair as well as by intrapair comparison, we have observed three sequence variants in a repetitive element that has peculiar capabilities with respect to the formation of unusual tertiary structures and might have a regulatory role. The integrity of this tertiary structure is protected by the rs62029389-rs62029390-rs62029391 haplotype GGG, associated as a risk allele with clinical severe disease manifestation.

### 3.4. Expression Levels of SCNN1B Depend on rs2303153

**Figure 2:**
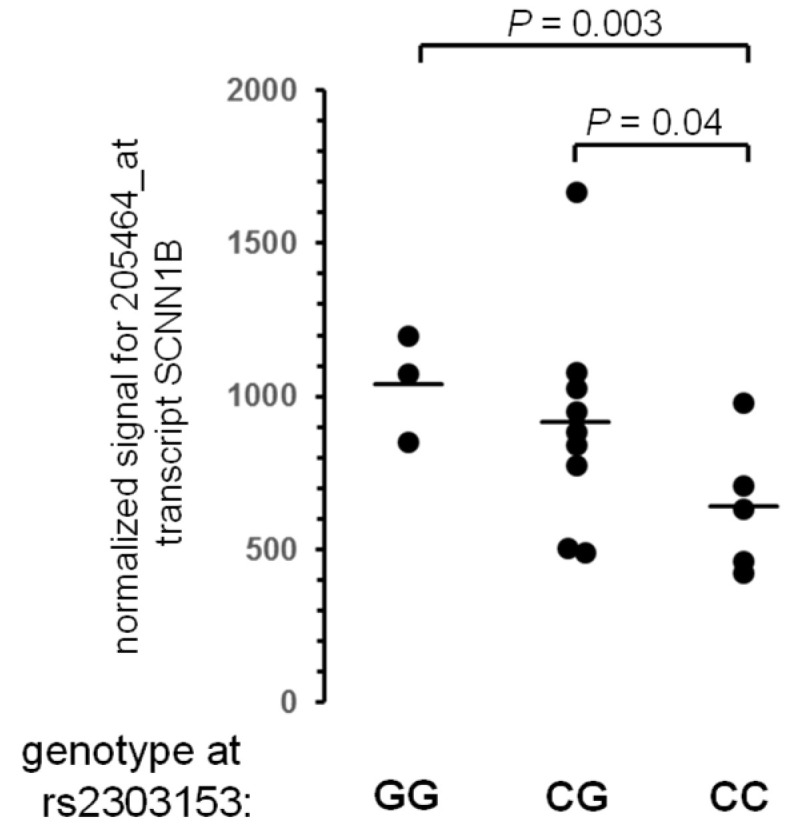
Expression of *SCNN1B* in rectal suction biopsies from F508del-*CFTR* homozygous CF patients. Data was retrieved for probe set 205464_at, representing *SCNN1B*, from global transcriptome analysis [35].

As rs2303153 resides in a putative regulatory element, we wanted to know if *SCNN1B* expression is influenced by the two contrasting rs2303153 alleles and reviewed transcriptome data on rectal suction biopsies [35], consisting predominantly of epithelia, for that purpose. *SCNN1B* expression was elevated among F508del-*CFTR* homozygotes who carry rs2303153-G in comparison to those patients who carry rs2303153-C (Figure 2, *p* = 0.003), substantiating that the mapped element on the genomic segment rs168748-rs2303153-rs4968000 determines *SCNN1B* expression in epithelia. 

## 4. Discussion

It has been shown in murine models and among patients that the expression levels of *SCNN1B* need to be tightly controlled to prevent pathological conditions: Reduced expression of Scnn1b in mice causes pseudohypoaldosteronism [43]. *P. aeruginosa* infection in mice provokes decreased Scnn1b expression [44]. In contrast, an increased expression of Scnn1b in mice causes CF-like lung disease [11,45]. As SCNN1B is a lowly expressed gene, interference with SCNN1B gene regulation by naturally occurring polymorphisms such as rs2303153 is likely to affect ENaC function through perturbation of SCNN1B expression levels and/or transcript stability, which explains the role of SCNN1B as a modifier of cystic fibrosis disease severity. Thus, our data on rs2305153-dependent SCNN1B expression in intestinal epithelia (Figure 2) underline that variants in regulatory elements can translate to clinically meaningful perturbations of epithelial ion homeostasis.

Naturally, such functional effects can only be recognized by an association study on patient populations when a sufficient power is generated due to the study design. As such, the analysis of sib pairs with extreme clinical phenotypes has been advocated as an ideal setting [28,29] to detect genetic modifiers as many non-inherited factors are shared between siblings of a pair, thus reducing the influence of a major non-genetic confounding variable in cystic fibrosis [46].

Furthermore, for a disease such as cystic fibrosis for which symptomatic therapy has considerably shaped survival in the last century [47], it is worth to notice that environmental variables are non-comparable between patients from different birth cohorts. For instance, epidemiological data on CF birth cohorts from the UK during the time period 1977–1985 reported a 50% survival at 19 years of age [47]. Currently, a median age of survival is estimated at 52 years for all European patients [46]. In other words, the improvements in therapeutic possibilities over the last decades have resulted in an incomparability of patients recruited into a cross-sectional study design and mandates the birth year to be taken into account.

In genetic research, changes in therapeutic approaches translate to a survivor bias that is observed among genetic data: in a cross-sectional recruitment, only patients who have survived until enrollment are being genotyped, whereby variants at genes that promote survival will be enriched among early-born patients who have survived [23,26]. This inherent challenge strongly cautions against genetic data obtained from cystic fibrosis patient populations recruited cross-sectionally without appropriate approaches to control for each patient’s respective birth cohort. Furthermore, this aspect might explain why, even if two studies agree on a particular candidate gene as a genetic modifier in cystic fibrosis [22,48], the same two studies disagree with respect to the annotation of the risk allele [22,48].

In conclusion, in a study designed to stringently minimize the effect of non-inherited environmental factors, we could identify a regulatory element at *SCNN1B* by fine-mapping (Pbest = 0.0177). We consistently observed the risk allele rs2303153-C and the contrasting benign allele rs2303153-G in three patient cohorts taking into account the survivor effect by stratifying the analysis for birth cohorts in a subsample of unrelated F508del-*CFTR* homozygotes. Furthermore, expression levels of *SCNN1B* were associated with rs2303153 genotype in intestinal epithelia (*p* = 0.003), corroborating our cohort findings. Our data demonstrate that the well-established biological role of *SCNN1B* can be recognized by an association study on informative endophenotypes in the rare disease cystic fibrosis and calls attention to the hitherto under-appreciated power of association studies obtained from small, albeit carefully characterized patient populations to yield reproducible, biologically meaningful results.

## Figures and Tables

**Table 1:** Variants observed on contrasting haplotypes identified after Sanger resequencing of the 3.000 bp genomic fragment defined by rs168748 to rs4968000.

Haplotype rs168748-rs2303153-rs4968000	Associated with Disease Manifestation:	rs168748 *	rs250570	rs62029389	rs62029390	rs62029391	rs61379932	rs2303153 *	rs4968000 *	rs3743966
CCA	severe	C	C	G	G	G	G	C	A	T
TCA	severe	T	G	G	G	G	G	C	A	T
TGC	mild	T	G	A	T	A	A	G	C	A
TGA	mild	T	C	A	T	A	A	G	A	A
Different when comparing CCA and TCA with TGC and TGA?		no	no	yes	yes	yes	yes	yes	no	yes

* rs168748-rs2303153-rs4968000 were used to map the fragment associated with disease severity and define the contrasting haplotypes CCA and TCA (associated with severe disease) as well as TGC and TGA (associated with mild disease). rs2303153 is not in LD with the neighboring markers rs168748 located 2 kb on the 5′ side (*r*² = 0.086; D’ = 0.341) and rs4968000 located 1 kb on the 3′ side (*r*² = 0.070; D’ = 0.277).

**Table 2:** Allele frequencies for rs2303153 in mildly and severely affected cystic fibrosis F508del-*CFTR* homozygous patients.

Allele at rs2303153	IntERpair Comparison on Sibling Pairs		IntRApair Comparison on Sibling Pairs		Unrelated Patients Stratified by Birth Cohort	
	Mildly Affected Sibling Pairs (CON+) 11 Pairs	Severely Affected Sibling Pairs (CON-) 10 Pairs	Mildly Affected Sib (DIS+) of Discordant Pairs 14 Patients	Severely Affected Sib (DIS-) of Discordant pairs 14 Patients	Born 1959-1977 * 34 Patients	Born 1978-1994 * 33 Patients
rs2303153-G	0.577	0.283	0.579	0.395	0.54	0.39
rs2303153-C	0.423	0.717	0.421	0.605	0.46	0.61
	Praw = 0.0177; Pcorr = 0.0528		Praw = 0.04024; Pcorr = 0.05		*p* = 0.084	

* for the unrelated patients from the CF clinic in Hannover, lung function was assessed during the first two years of data reporting by FEV1, transformed to normalized values based on global lung initiative calculation. For the patients of whom data on rs2303153 is displayed in this table, FEV1 in this initial reporting period was above median within each of the subgroups stratified by year of birth. For unrelated patients from this cohort who had a below-average lung function, pooled irrespective of their year of birth, the observed of genotype distribution at rs2303153 deviated from the genotype distribution expected under assumption of the Hardy-Weinberg-law (*p* = 0.05; excess of homozygotes for rs2303153-G observed).

## Data Availability

Primary data will be shared with interested parties upon reasonable request. The transcriptome data on rectal suction biopsies is available at the GEO database (GSE1556829).

## Supplementary Materials

The following are available online at https://www.mdpi.com/article/10.3390/genes12101554/s1, Supplementary Figure S1: Complex repetitive element surrounding markers rs62029389, rs62029390 and rs62029391, Supplementary Figure S2: Predicted site for the general transcription factor TFII-I altered by SNPs rs62029391, rs62029390 and rs62029389.
