# Supplementary material for: Consistent Assignment of Risk and Benign Allele at rs2303153 in the CF Modifier Gene SCNN1B in Three Independent F508del-CFTR Homozygous Patient Populations
Source: Genes (Basel). 2021 Sep 29;12(10):1554. doi: 10.3390/genes12101554 (PMC8535344; doi:10.3390/genes12101554)
Supplement: Supplementary file 1 [file genes-12-01554-s001.zip › genes-1387623-supplementary.pdf]

Supplementary Material for:

Consistent assignment of risk and benign allele at rs2303153 in the CF modifier gene SCNN1B in three independent F508del-CFTR homozygous patient populations

Frauke Stanke, Tim Becker, Haide Susanne Ismer, Inga Dunsche, Silke Hedtfeld, Julia Kontsendorn, Anna-Maria Dittrich, Burkhard Tümmler

1 Genotyping data set

The genotyping data set described here has been employed within two projects:

1.) this work

and within

2.) Becker T, Pich A, Tamm S, Hedtfeld S, Ibrahim M, Altmüller J, Dalibor N, Toliat MR, Janciauskiene S, Tümmler B, Stanke F. Genetic information from discordant sibling pairs points to ESRP2 as a candidate trans-acting regulator of the CF modifier gene SCNN1B. Sci Rep. 2020 Dec 31;10(1):22447. doi: 10.1038/s41598-020-79804-y. [1]

These two manuscripts describe two different elements within SCNN1B which are both of relevance to understand the CF modifying gene SCNN1B. In particular, Sci Rep. 2020 Dec 31;10(1):22447 [1] describes a regulatory element that manifest in conjunction with an interaction partner encoded in trans and was recognized by comparing discordant sibling pairs to all concordant sibling pairs. In contrast, this work describes a regulatory element that can be recognized in cis, whereby mildly and severely affected patient pairs carry different SCNN1B genotypes.

2 Marker Genotyping

Genotyping of SNPs by PCR-RFLP (Supplementary Table 1) in a 30µl reaction volume was done using 50 ng of genomic DNA, 0.4 U of Taq DNA Polymerase, 15 pmol of each primer, 6 nmol of each dNTP and experimentally predetermined amounts of MgCl2 (range: 50 nmol to 150 nmol), dimethylsulfoxide (up to 1µl) and betain (up to 30 nmol). The restriction digest to distinguish both alleles at a SNP site by PCR-RFLP was done on 10 to 15 µl of unpurified PCR product using 1 U of restriction enzyme. Incubation was done overnight for classical restriction enzymes such as BsaXI, HpyCH4IV or HpaII, but restricted to 2 h for time-saver enzymes provided by NE Biolabs such as BslI and DdeII.

SNPstream genotyping was carried out with the GenomeLab SNPstream high-throughput 48-plex genotyping platform from Beckman Coulter following manufacturer's instructions. Primers for the multiplex PCR and single-base extension (SBE) were designed with web-based software provided at http://www.autoprimer.com (Beckman Coulter inc. Fullerton, CA). To ensure genotyping quality, several controls were included to exclude mix-ups and other errors during genotyping. Thus, each 384-well plate contained 4 blanks to detect contamination with DNA and 4 randomly selected replicates, which are expected to yield identical genotypes for a given genetic variant. 48-plex PCR reactions were performed in 384-well plates (4titude, UK) in a volume of 5 µl using 4 ng of DNA, dNTP mix (90 µM each) , 0.5 U of AmpliTaq Gold (Perkin-Elmer, Wellesley, MA), 1 X PCR buffer II, 5mM Mg and the 48 PCR primers at a concentration of 50 nM each. Thermal cycling was performed in GeneAmp PCR system 9700 thermal cyclers (Applied Biosystems, Foster City, CA) using the following program: initial denaturation at 94°C for 1 min followed by 40 cycles of 94°C for 30 s, 55°C for 30 s, 72 °C for 1 min and 4°C final hold temperature. Following PCR, plates were centrifuged briefly and 3 µl of a 1:25 dilution of SBE Clean-Up Reagent (USB; Cleveland, OH) was added to each well. The plates were sealed and incubated for 30 min at 37 °C and at 96 °C for 10 min. After the purification step, the SBE reaction was performed using reagents and protocols specific to the SNPstream platform. In the last step every extension product was hybridized to the complementary oligonucleotides arrayed on the 384-well microplates (SNPware 48-plex Tag array plate) by incubation at 42°C for 2 hours with humidity close to 100%. The SNPware

plates were imaged after washing and drying with a two-laser, two-color charged couple device-based imager (GenomeLab SNPstream array imager). The 48 individual SNPs were identified by their position and fluorescent color in each well according to the position of the tagged oligonucleotides. Sample genotype data was generated on the basis of the relative fluorescent intensities for each SNP and electronically processed for graphical review.

Microsatellite markers were amplified in a 15 µl reaction volume using 50 ng of genomic DNA, 0.25 U of Taq DNA Polymerase, 12.5 pmol of unbiotinylated and 2.5 pmol of biotinylated primer, 3 nmol of each dNTP and experimentally predetermined amounts of MgCl2 (range: 25 nmol to 150 nmol), dimethylsulfoxide (up to 1 µl) and betain (up to 30 nmol). Biotinylated PCR products were separated and transferred onto a Hybond N+ positively charged nylon membrane (RPN303B, GE Healthcare Life Sciences, Little Chalfont, United Kingdom) by direct blotting electrophoresis (GATC, Konstanz, Germany). Signals were visualized using Blocking reagent (11096176001, Roche, Basel, Switzerland), Streptavidin-AP Conjugate (1189161001, Roche, Basel, Switzerland) and CDPstar (T2305, Applied Biosystems, Waltham, Massachusetts, USA).

Signals generated by GelRed were visualized on a GelDoc XR Molecular Imager (Bio-Rad, Hercules, California, USA). CDPstar signals from microsatellite genotyping and luminol signals generated for EMSA-PSeq experiments (see below) were visualized on a DNR-MF-ChemiBIS 3.2 Bio-Imaging System (Berthold Technologies, Bad Wildbad, Germany).

3 Supplementary Figures

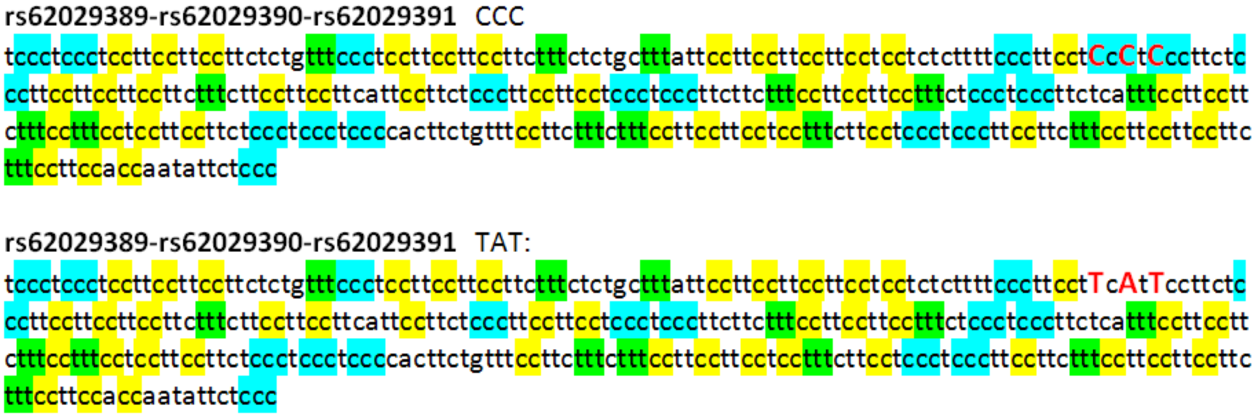

Supplementary Figure S1. Complex repetitive element surrounding markers rs62029389, rs62029390 and rs62029391

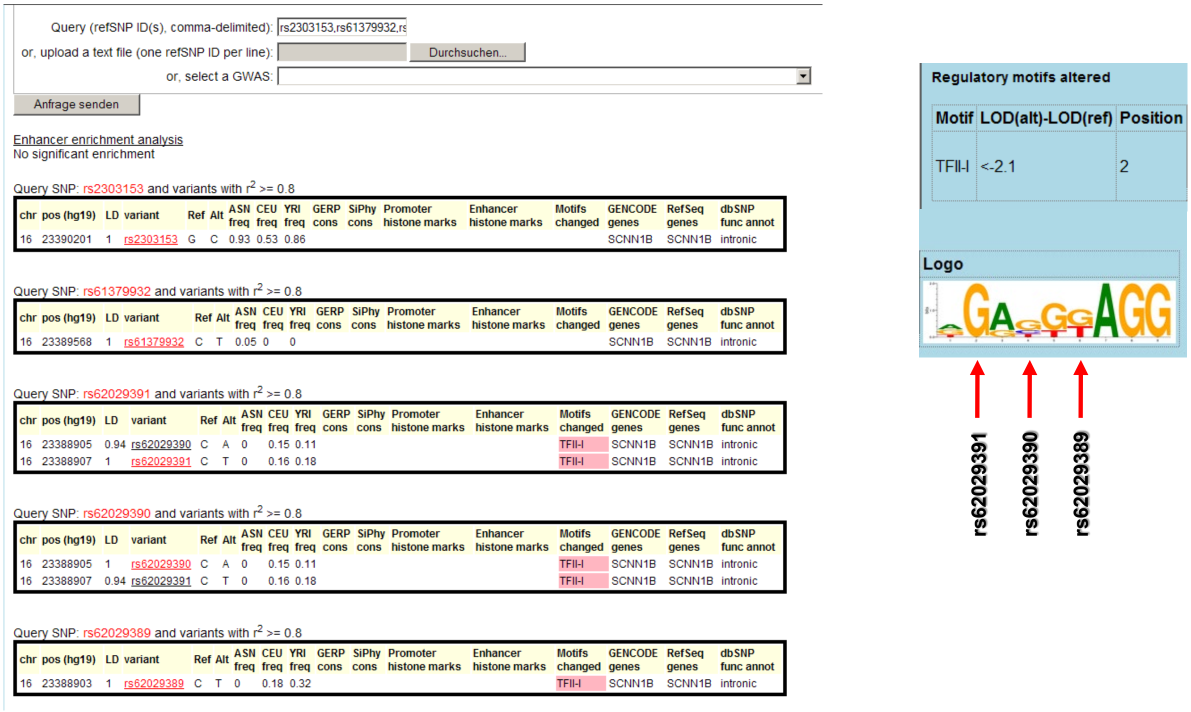

Query (refSNP ID(s), comma-delimited): rs2303153,rs61379932,rs
or, upload a text file (one refSNP ID per line): Durchsuchen...
or, select a GWAS:
Anfrage senden

Enhancer enrichment analysis
No significant enrichment

Query SNP: rs2303153 and variants with r^2 >= 0.8

chr	pos (hg19)	LD	variant	Ref	Alt	ASN freq	CEU freq	YRI freq	GERP cons	SiPhy cons	Promoter histone marks	Enhancer histone marks	Motifs changed	GENCODE genes	RefSeq genes	dbSNP func annot
16	23390201	1	rs2303153	G	C	0.93	0.53	0.86						SCNN1B	SCNN1B	intronic

Query SNP: rs61379932 and variants with r^2 >= 0.8

chr	pos (hg19)	LD	variant	Ref	Alt	ASN freq	CEU freq	YRI freq	GERP cons	SiPhy cons	Promoter histone marks	Enhancer histone marks	Motifs changed	GENCODE genes	RefSeq genes	dbSNP func annot
16	23389568	1	rs61379932	C	T	0.05	0	0						SCNN1B	SCNN1B	intronic

Query SNP: rs62029391 and variants with r^2 >= 0.8

chr	pos (hg19)	LD	variant	Ref	Alt	ASN freq	CEU freq	YRI freq	GERP cons	SiPhy cons	Promoter histone marks	Enhancer histone marks	Motifs changed	GENCODE genes	RefSeq genes	dbSNP func annot
16	23388905	0.94	rs62029390	C	A	0	0.15	0.11					TFII-I	SCNN1B	SCNN1B	intronic
16	23388907	1	rs62029391	C	T	0	0.16	0.18					TFII-I	SCNN1B	SCNN1B	intronic

Query SNP: rs62029390 and variants with r^2 >= 0.8

chr	pos (hg19)	LD	variant	Ref	Alt	ASN freq	CEU freq	YRI freq	GERP cons	SiPhy cons	Promoter histone marks	Enhancer histone marks	Motifs changed	GENCODE genes	RefSeq genes	dbSNP func annot
16	23388905	1	rs62029390	C	A	0	0.15	0.11					TFII-I	SCNN1B	SCNN1B	intronic
16	23388907	0.94	rs62029391	C	T	0	0.16	0.18					TFII-I	SCNN1B	SCNN1B	intronic

Query SNP: rs62029389 and variants with r^2 >= 0.8

chr	pos (hg19)	LD	variant	Ref	Alt	ASN freq	CEU freq	YRI freq	GERP cons	SiPhy cons	Promoter histone marks	Enhancer histone marks	Motifs changed	GENCODE genes	RefSeq genes	dbSNP func annot
16	23388903	1	rs62029389	C	T	0	0.18	0.32					TFII-I	SCNN1B	SCNN1B	intronic

Supplementary Figure S2. Predicted site for the general transcription factor TFII-I altered by SNPs rs62029391, rs62029390 and rs62029389. Data was analysed with Haploreg (http://compbio.mit.edu/HaploReg; [2]; accessed in 03/2012).

4 References

1. Becker, T.; Pich, A.; Tamm, S.; Hedtfeld, S.; Ibrahim, M.; Altmüller, J.; Dalibor, N.; Toliat, M.R.; Janciauskiene, S.; Tümmler, B.; et al. Genetic Information from Discordant Sibling Pairs Points to ESRP2 as a Candidate Trans-Acting Regulator of the CF Modifier Gene SCNN1B. *Sci Rep* **2020**, *10*, 22447, doi:10.1038/s41598-020-79804-y.
2. Ward, L.D.; Kellis, M. HaploReg: A Resource for Exploring Chromatin States, Conservation, and Regulatory Motif Alterations within Sets of Genetically Linked Variants. *Nucleic Acids Res.* **2012**, *40*, D930-4, doi:10.1093/nar/gkr917.
